# A bench-to-data analysis workflow for respiratory syncytial virus whole-genome sequencing with short and long-read approaches

**DOI:** 10.1186/s13073-025-01597-4

**Published:** 2026-01-27

**Authors:** Adrián Gómez-Del Rosario, Adrián Muñoz-Barrera, Julia Alcoba-Florez, Diego García-Martínez de Artola, Nora Rodríguez-García, Jose Miguel Lorenzo-Salazar, Rafaela González-Montelongo, Carlos Flores, Laura Ciuffreda

**Affiliations:** 1 Research Unit, Hospital Universitario Nuestra Señora de Candelaria, Instituto de Investigación Sanitaria de Canarias (IISC), Santa Cruz de Tenerife, Spain; 2 CIBER de Enfermedades Respiratorias (CIBERES), Instituto de Salud Carlos III, Madrid, Spain; 3 Genomics Division, Instituto Tecnológico y de Energías Renovables (ITER), Santa Cruz de Tenerife, Spain; 4 Microbiology Unit, Hospital Universitario Nuestra Señora de Candelaria, Instituto de Investigación Sanitaria de Canarias (IISC), Santa Cruz de Tenerife, Spain; 5 Department of Clinical Sciences, University Fernando Pessoa Canarias, Las Palmas de Gran Canaria, Spain

**Keywords:** Respiratory syncytial virus, Genomic surveillance, Short-read sequencing, Nanopore

## Abstract

Genomic surveillance of respiratory syncytial virus (RSV) enables detecting and monitoring of circulating lineages and the emergence of amino acid substitutions affecting transmission, severity, and treatment. We present a comprehensive workflow for RSV whole-genome sequencing, from sample processing to lineage classification. Tiling amplicon protocols adapted for Illumina and Oxford Nanopore Technologies sequencing approaches and an in-house bioinformatic pipeline were developed for the analysis of samples. We validated the workflow on clinical samples, and identified RSV sequences harbouring substitutions associated with monoclonal antibody resistance and the presence of coinfections.

**Supplementary Information:**

The online version contains supplementary material available at 10.1186/s13073-025-01597-4.

## Background

Respiratory syncytial virus (RSV) has a major impact on public health as it is the leading cause of acute lower respiratory tract infections in infants and children under two years of age [1]. There are two main antigenic subgroups of RSV (RSVA and RSVB), defined by the G glycoprotein, that co-circulate globally, with the predominance of one or the other depending on the different epidemic seasons. In the majority of worldwide RSV infections, patients are asymptomatic or have mild symptoms [2, 3]. However, some patients can develop severe illness that can lead to hospitalization [4]. RSV can also severely affect adults, especially those with comorbidities, immunocompromised, or the elderly [5]. In Spain, two out of every 100 children under two years of age are hospitalized every year, with an estimated annual cost to the National Health System of 50 million euros [6, 7]. On a global scale, the annual economic impact of RSV is estimated to be ~ 4.82 billion euros, considering only children under five years of age [8]. Substantial efforts have been made to develop an immunoprophylactic treatment strategy based on vaccination or neutralising monoclonal antibodies (mAb). In March 2023, nirsevimab, a long-acting anti-RSV fusion protein mAb, was included in the immunization schedule of some Spanish regions, such as Galicia [9] and the Canary Islands, and then into the rest of the country [10]. By now, rare, nirsevimab-resistant RSV lineages have been reported [9].

Genomic surveillance of RSV is crucial for the identification and monitoring of the circulating lineages because it provides insight into the emergence of amino acid substitutions associated with resistance to treatments and vaccines, as well as information on viral evolution and epidemiology. Few protocols have been established for this purpose, most of which target only specific RSV regions, such as the *G* and *F* genes, which do not represent the total viral genome diversity [11–13]. In addition, most available workflows for genomic surveillance of RSV have been optimised for a single sequencing technology, either Oxford Nanopore Technologies (ONT) or Illumina [14–19], while only a few have been designed for both sequencing platforms [20–22], primarily focusing on either the experimental or the bioinformatics aspects. For example, Dong et al. developed one experimental protocol suitable for both sequencing technologies, but did not provide any detail on the bioinformatics pipeline used [20]. In another study, Doosba et al., developed RSV-GenoScan which enables automatization of the RSV whole-genome sequencing data analysis, without performing the optimization of the experimental protocol [21]. Here, we have adapted an RSV whole-genome tiling amplicon sequencing protocol, originally developed for Illumina sequencing [23], to nanopore sequencing using ONT and developed an in-house bioinformatic pipeline for the analysis of the genomic data generated by the two technologies [24]. We have then tested and compared the results obtained by this novel workflow using clinical samples processed by both sequencing technologies. To the best of our knowledge, this is the first time a direct comparison of RSV sequencing results obtained by Illumina and ONT platforms is extensively performed. Furthermore, our study represents one of the few RSV genomic surveillance efforts in Spain [23, 25], and the first one in the Canary Islands.

## Methods

### Study design and cohort

The study, which was exempt from obtaining informed consent from patients, was approved by the Research Ethics Committee of the Complejo Hospitalario Universitario de Canarias review board (reference number: CHUNSC_2024_48). Samples were obtained from patients who required hospital care (hospitalized, admitted to the emergency room or outpatients) with symptoms compatible with a respiratory viral infection or from immunosuppressed patients screened on admission. After reverse-transcription quantitative PCR (RT-qPCR) used for routine diagnosis, convenience sample selection was performed based on cycle threshold (Ct; <18, 18–20, 20–22, 22–24, 24–26, ≥ 26) to ensure balanced representation across ranges. A total of 176 RSV positive nasopharyngeal swab samples, collected from 173 patients in Tenerife (Canary Islands, Spain), were chosen for this study and sequenced using both Illumina and ONT sequencing technologies. Table 1 details the cohort characteristics corresponding to the patients for whom data was generated in this study. The study period encompassed the 2022–2023 RSV season (October 2022, epidemiological week 42 to March 2023, epidemiological week 10) and the 2023–2024 RSV season (June 2023, epidemiological week 25 to February 2024, epidemiological week 7).

### Sample collection from patients and diagnosis

Nasopharyngeal swab samples were collected in Universal Transport Medium^®^ for viruses (COPAN, Brescia, Italy) with minitipped flocked nylon FLOQSwab (COPAN) and sent to the Microbiology Unit of the University Hospital Nuestra Señora de Candelaria (Santa Cruz de Tenerife, Spain) for routine molecular diagnosis. RSV was detected by RT-qPCR using one of the following two commercial kits: the Xpert^®^ Xpress CoV-2/Flu/RSV plus [Cepheid, Sunnyvale, CA, USA] and the Alinity m Resp-4-plex assay [Abbott Laboratories, Chicago, IL, USA]. Among the RSV positive samples with Ct < 30 eligible for sequencing, RNA was isolated from nasopharyngeal swabs using MagNA pure 96 DNA and Viral NA Small Volume kit (Roche Life Science [Basel, Switzerland]) with a starting volume of 200 µl and a final elution volume of 50 µl.

### Library preparation and sequencing

For Illumina sequencing, the protocol for RSV tiling genome amplification and the primer scheme was previously described [23]. Briefly, the primer scheme consists of a total of 39 primers, divided into two pools of amplicons (19 primers in pool 1 and 20 primers in pool 2). The resulting amplicons span the complete RSV genome, with 10 amplicons for RSVA (with an average length of 1,767 bp, range: 1,556–1,930 bp) and 10 amplicons for RSVB (with an average length of 1,742 bp, range: 1,413–1,960 bp). Libraries were prepared using the Illumina CovidSeq Test (Illumina Inc., San Diego, CA, USA), quantified using Qubit™ dsDNA HS Assay Kit (Invitrogen, Eugene, CA, USA) and checked on a 4200 TapeStation (Agilent Technologies, Santa Clara, CA, USA). Sequencing was performed on an Illumina NextSeq 550 system with the High Output Kit v2.5 (150 cycles) in four runs, using 1.4 pM as the final concentration, and spiked with 1% PhiX Control v3 (Illumina Inc.). A total of 1,452 samples were multiplexed in these runs, of which only 176 RSV-positive samples were included in the present study. The remaining samples corresponded to a mixture of other respiratory viruses processed within the same sequencing batches.

For ONT sequencing, a two-step RT-PCR was performed using the Midnight RT PCR Expansion kit (ONT, Oxford, UK). For each sample, 2 µl of the Luna Script RT Super Mix were added to 8 µl of RNA. The RT-step was performed using the following conditions: 25 °C for 2 min, 55 °C for 10 min and 95 °C for 1 min. cDNA was then amplified in two PCR reactions, one for each primer pool, using the same primers employed for Illumina sequencing [23]. PCR conditions consisted of an initial denaturation step at 98 °C for 2 min followed by 35 cycles of a denaturation step at 98 °C for 15 s, and a combined annealing and extension step at 63 °C for 7 min. For each sample, 6.25 µl of Q5 HotStart Master Mix, 0.05 µl of primer pool 1 or 2 (100 µM), 2.5 µl of cDNA, and 3.7 µl of nuclease-free water were used. Libraries were prepared using the Rapid Barcoding Kit 96 (ONT) with 47 samples multiplexed in each run and sequencing was performed using four R9.4.1 flow cells in a MinION Mk1B or a GridION X5 (ONT) device for at least 48 h. Basecalling was performed using the MinKNOW v24.02.16 and the integrated basecaller Dorado v7.3.11 with the super-accuracy basecalling model.

### Bioinformatic pipeline for sequencing data analysis

Using Nextflow [26] and following nf-core [27] guidelines and templates as framework, a full in-house bioinformatic pipeline was developed integrating state-of-the-art tools (Additional file 1: Table S1) to generate consensus sequences of RSV viruses using Illumina or ONT sequencing data [24].

**Fig. 1:**
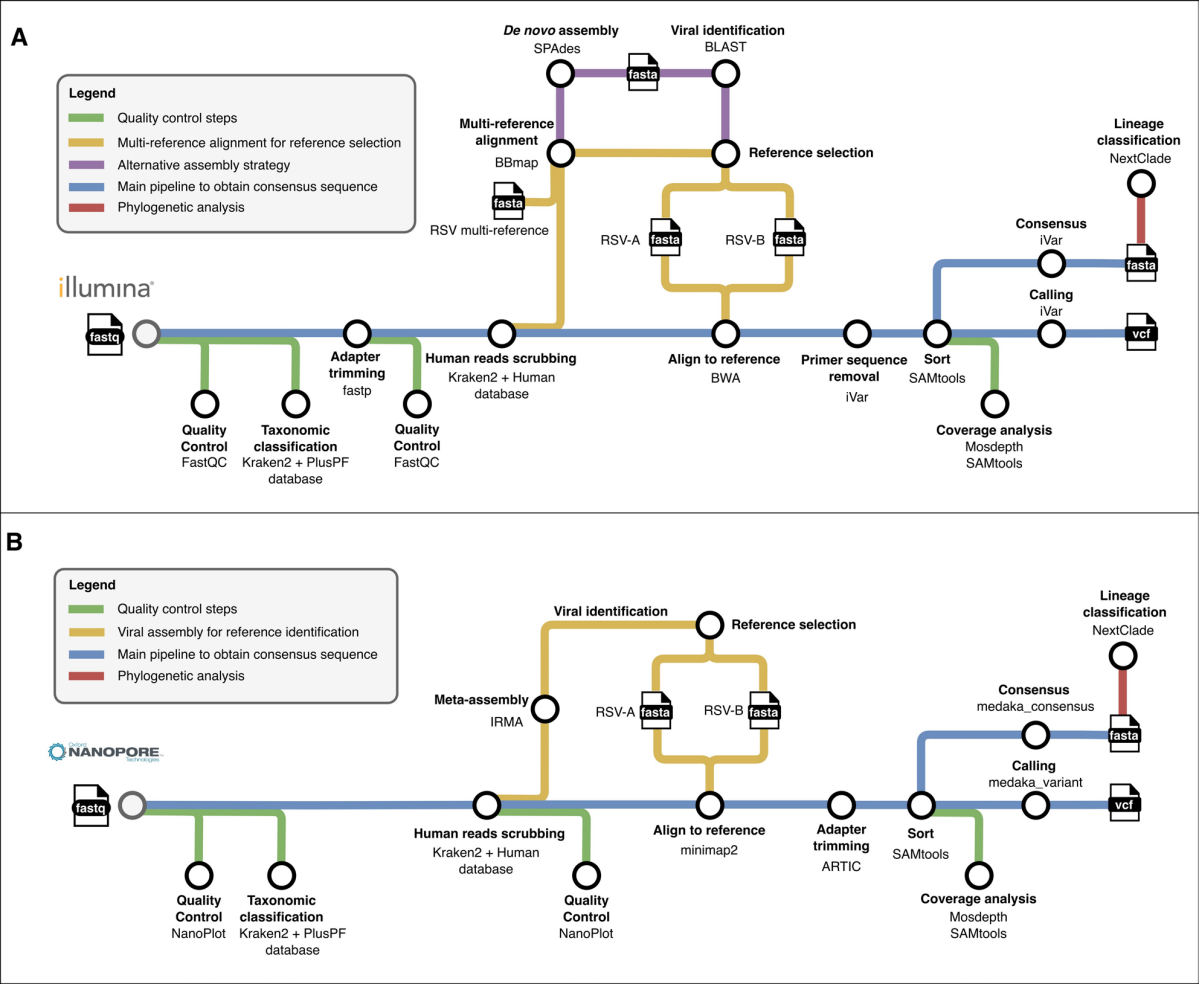
Bioinformatic pipeline to obtain the RSV consensus sequences from Illumina (**A**) or Oxford Nanopore Technologies (**B**) sequencing data

To generate the consensus sequences of RSV viruses using Illumina sequencing (Fig. 1A), the developed pipeline starts with a quality control step using FastQC [28] to assess sequence quality metrics. To confirm the presence of RSV in the samples, a taxonomic classification of reads is performed using Kraken2 [29] with the PlusPF database. Adapter sequences are removed from the reads using fastp [30], and human-host reads are scrubbed by means of Kraken2 with the human-only database. The reference sequences *hRSV/A/England/397/2017* and *hRSV/B/Australia/VIC-RCH056/2019*, included in a multi-reference FASTA file, are used to identify the most appropriate RSV reference for downstream analysis. This is achieved by performing a multiple sequence alignment (MSA) of the reads using BBmap [31]. If the MSA step fails, an alternative assembly strategy using SPAdes [32] is performed, followed by a contig classification step using BLAST [33] to assign the sample to the correct antigenic subgroup (RSVA/RSVB) and select the appropriate viral reference sequence. The remaining viral reads after scrubbing are mapped to the selected reference genome using BWA [34] and primer adapters are trimmed using iVar [35]. Genome-wide breadth coverage and specific coverage of the *G* and *F* genes are assessed using Mosdepth [36] and SAMtools [37] to report coverage distribution. Mutations and consensus sequences are obtained by piping SAMtools pileup with the iVar derived consensus output, as described elsewhere [38], using a minimum depth of 5 reads and a minimum base quality threshold of 10. Finally, consensus sequences are submitted to the Nextclade [39] web version for qualitative mutation calling, sequence quality assessments, and lineage assignment after phylogenetic placement.

Given the differences in read lengths and error profiles associated with Illumina and ONT raw data, specific tools and quality control steps are incorporated into the ONT pipeline to ensure accurate viral genome reconstruction (Fig. 1B). Quality control is performed using NanoPlot [40]. Then, taxonomic classification and host-read removal are performed using Kraken2 with the PlusPF database and the human-only database, respectively. The remaining non-human reads are subjected to a meta-assembly step by means of IRMA [41], using the provided RSV-specific module, to select the most appropriate RSV reference genome for downstream analyses. The de-hosted viral reads are subsequently mapped to the selected RSV reference using minimap2 [42], and adapter trimming is conducted with the align_trim tool from the ARTIC pipeline [43]. Coverage analysis is performed using the same methods described for the Illumina pipeline. Finally, variant calling and consensus sequence generation are carried out using Medaka [44]. Unlike iVar, Medaka does not allow tuning of depth or quality thresholds; instead, it applies a neural network–based polishing model to directly infer the consensus sequence from read alignments. In this study, we used the model r941_min_sup_variant_g507, which is optimized for ONT R9.4.1 flow cells. Qualitative mutation calling, sequence quality assessments, and lineage assignment are performed using the NextClade web version.

All datasets were processed using the TeideHPC cluster infrastructure [45] and a local workstation running Rocky Linux 8 equipped with 2 Intel^®^ Xeon^®^ Gold 6252 CPUs at 2.10 GHz and 376 GB of RAM.

### Analysis of coinfections

To evaluate potential coinfections in some samples, an RT-qPCR using the Allplex™ Respiratory Panel 1 kit (Seegene, Seoul, South Korea) was performed, which enables differentiation between RSVA and RSVB infections. When low concordance for the lineage-defining mutations could not be explained by a loss of coverage in the sequence, alignment BAM files were manually inspected with Integrative Genome Viewer (IGV) v2.17.4 ​​ [46]. ViralFlow (v1.1.1) [47] was used to confirm the presence of intra-host viral lineages of the same antigenic subgroup.

### Amino acid substitutions associated with the efficacy of neutralizing monoclonal antibodies

For the analysis of the amino acid substitutions related to reduced efficacy to mAbs, we searched for amino acid changes in the nirsevimab binding site (site Ø of F protein, amino acid residues 62–69 and 196–212) and the palivizumab binding site (site II of F protein, amino acid residues 262–275), as previously described [48]. Only samples that had both binding sites covered in the sequence were included in this analysis.

### Phylogenetic analysis

Phylogenetic trees were constructed to place the resulting lineages in the epidemiological context and to evidence that the genomic sequences generated in this study were representative of circulating RSVA and RSVB viruses. Sequences were uploaded to Nextclade [39] and rooted to the reference sequence EPI_ISL_412866 for RSVA and EPI_ISL_1653999 for RSVB. The resulting JSON files were downloaded and submitted to Auspice [49] and then manually modified in Inkscape v.1.2.2 to improve visualization.

### Statistical analysis

To assess the differences between the Illumina and ONT sequencing strategies, we compared the genomic breadth of coverage, defined as the percentage of the consensus sequence covered, the lineage assigned to each consensus sequence by Nextclade, and the concordance in detecting the lineage-defining mutations following [50]. For the analysis of the genomic breadth of coverage, a chi-square test was used. Logistic regression modelling was performed to assess whether different factors (depth of coverage, Ct, patient sex and age, RSV subgroup and RSV lineage assignment) affected the sequencing success, defined by achieving at least 80% of the viral genome. The best-fitting and most parsimonious model was selected based on the Akaike information criteria. To compare the lineage-defining mutations, the concordance found by the two technologies was calculated for each mutation as the number of matches divided by the number of sequences assigned to the lineage. Data analysis was performed using R v4.3.2 [51]. Statistical significance was established at *p* < 0.05.

## Results

### Epidemiological and comparative analysis of RSV results by sequencing technology

A total of 176 samples were collected from 173 patients. Of these, 84 (48.55%) were male and 89 (51.45%) were female. Most patients were children up to two years of age (48.56%) and elderly over 60 (38.15%). Twenty-seven (15.6%) cases required hospitalization (Table 1). Defining reinfection as the detection of two RSV-positive samples collected at least 30 days apart, two cases of reinfection were observed among the 173 patients.

**Table 1:** Demographic data of the patients with symptoms compatible with a viral infection included in the study (*n* = 173)

Variable		Count, *n* (%)
Sex, male		84 (48.55)
Age interval, years	0–2	84 (48.55)
	3–5	3 (1.73)
	6–60	20 (11.56)
	> 60	66 (38.15)
Patient status	Hospitalized	27 (15.61)
	Non hospitalized^$^	146 (84.39)

^$^Patients admitted to the emergency room or outpatients

**Fig. 2:**
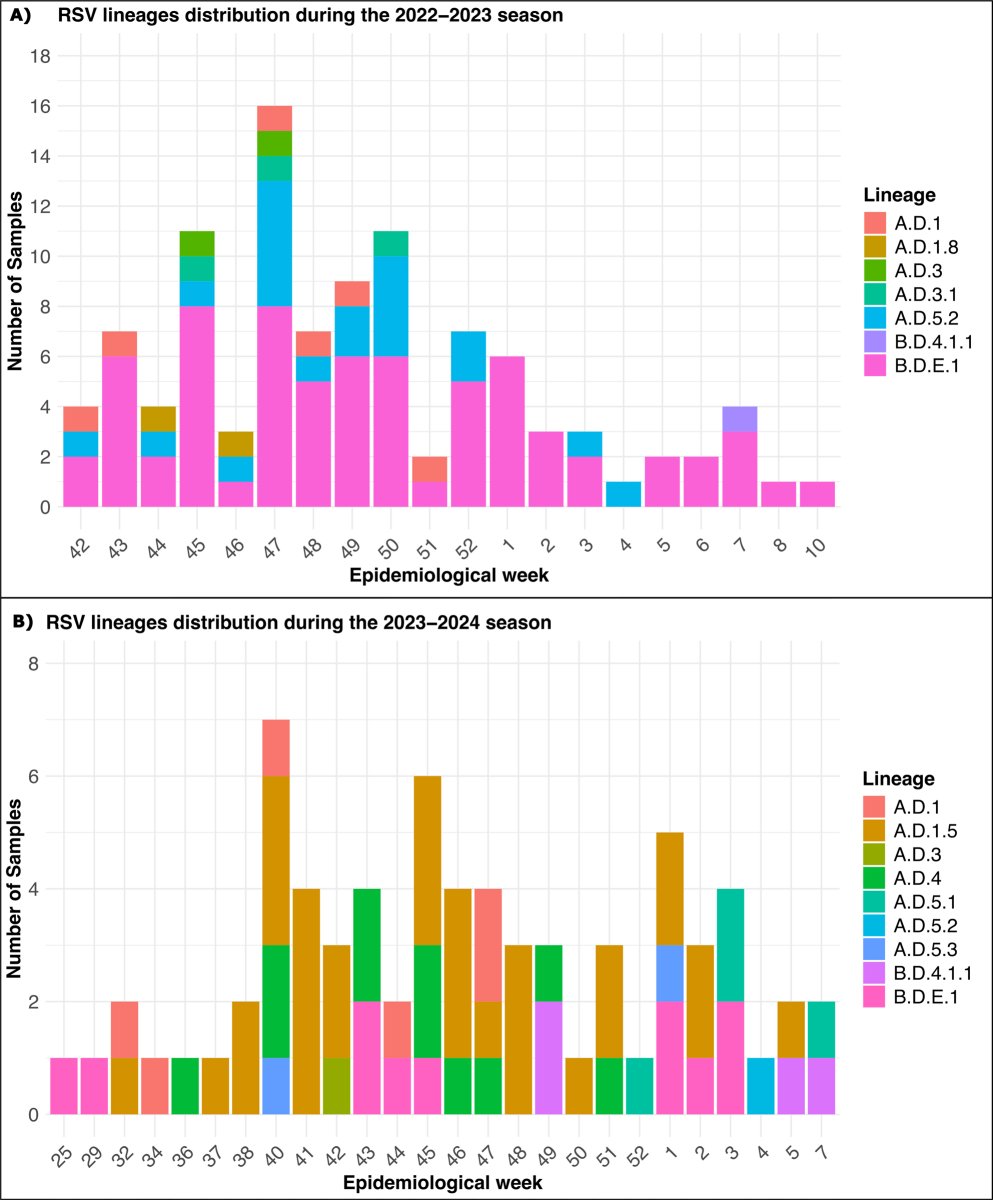
Nextclade lineage assignment series of RSV sequences from the 2022–2023 (**A**) and 2023–2024 (**B**) seasons

Out of the 176 samples collected for this study, in the 2022–2023 season (*n* = 105), RSVB lineages predominated with 68.6% of RSV infections attributed to this antigenic subgroup, most of them assigned to B.D.E.1 (Fig. 2A). In contrast, during the 2023–2024 season (*n* = 71), RSVA lineages predominated, representing 78.9% of cases, with the A.D.1.5 lineage being the most common (Fig. 2B). Phylogenetic analysis confirmed that the sequences collected in the Canary Islands are representative of those circulating worldwide during the two epidemic seasons covered by the study (Additional file 1: Fig. S1 and Fig. S2).

**Fig. 3:**
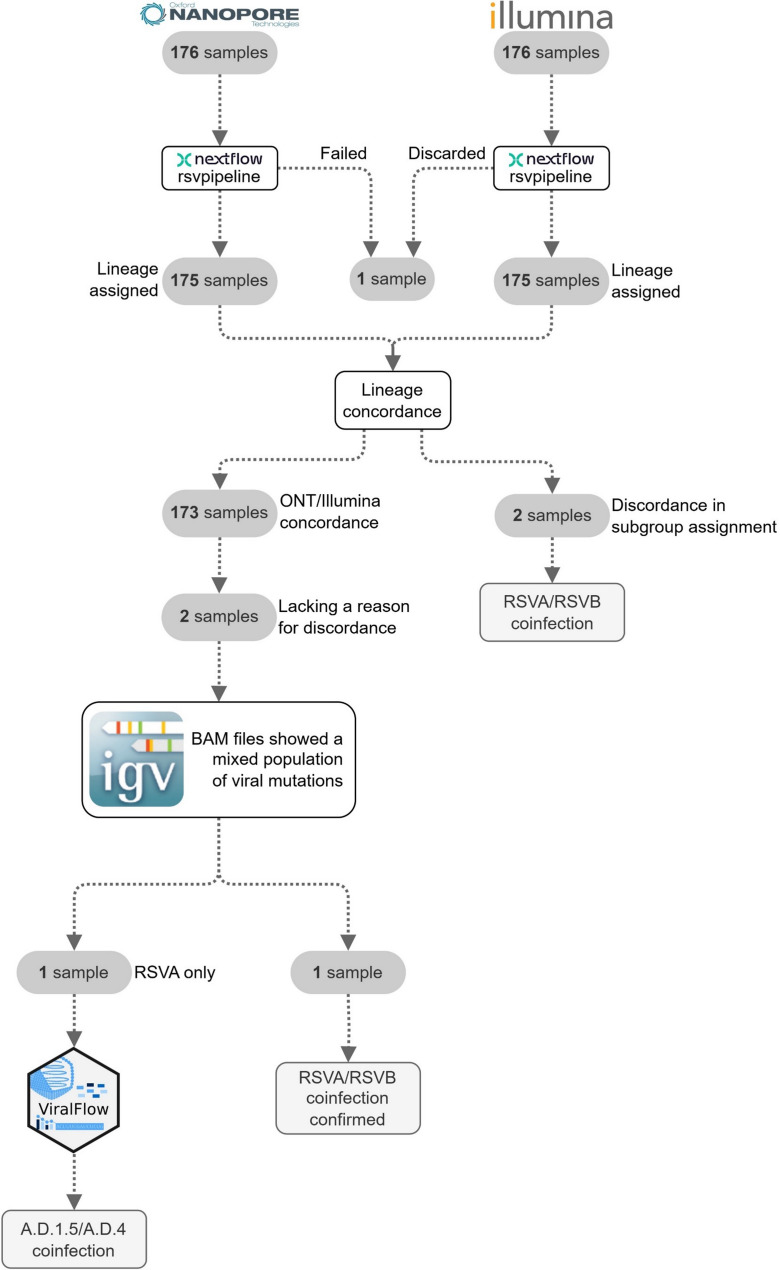
Flow chart of the analysis workflow

**Fig. 4:**
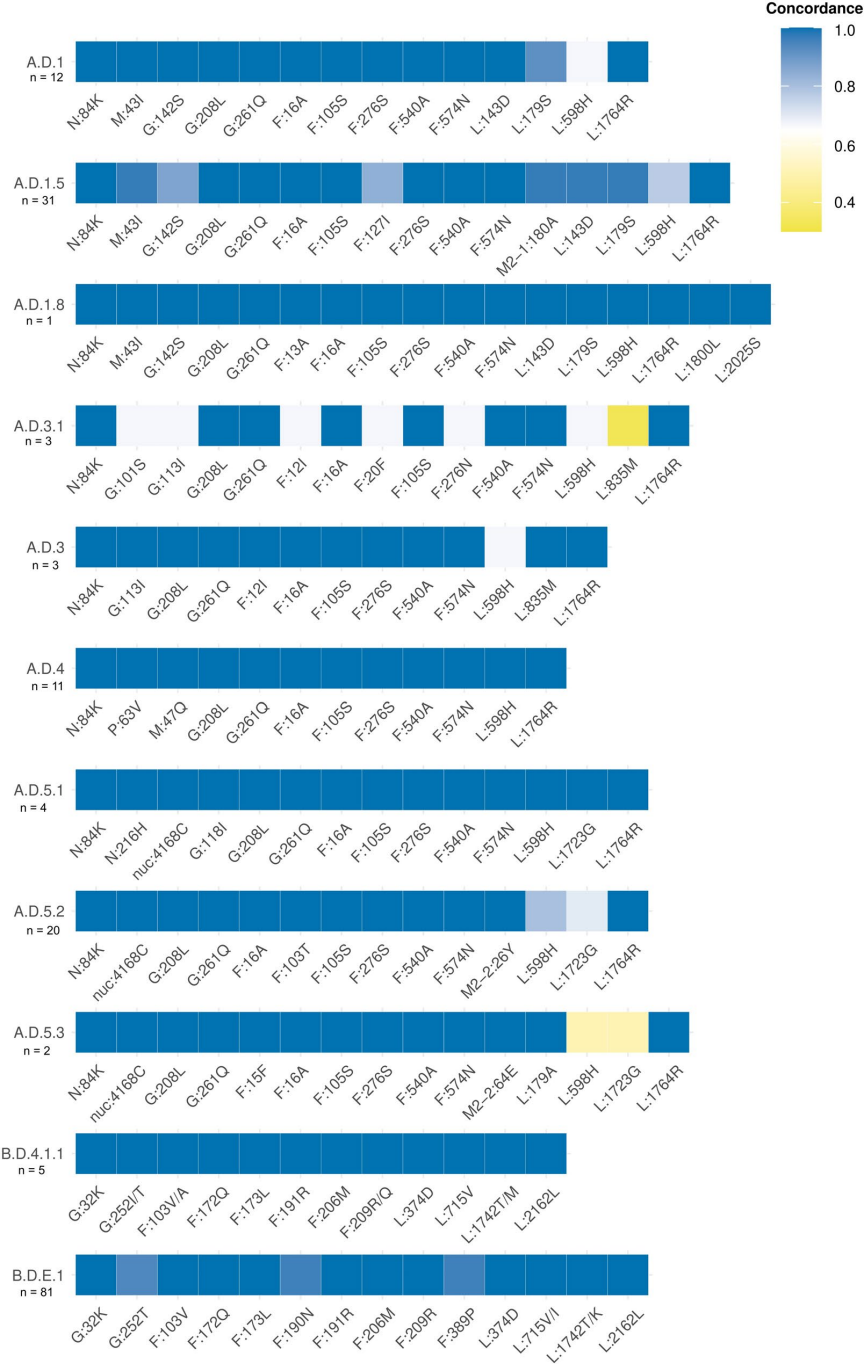
Heatmap of the Illumina and ONT concordance of the lineage-defining mutations, based on Goya et al. [50]. Only samples that are assigned to the same lineage using Illumina and ONT are represented

A total of 176 samples were sequenced using the previously described Illumina and ONT protocols. The median number of reads obtained was 2.1 million reads (range 0.81–5.0.81.0 million reads) for Illumina and 95.2 thousand reads (range 1–397 thousand reads) for ONT (Additional file 2: Table S2). One sample failed to complete the pipeline due to the low number of reads obtained by ONT and was discarded from the comparative analyses. The fallback assembly strategy using SPAdes for the reference selection, implemented only for Illumina-sequenced samples, was triggered only for one sample showing a high Ct value (28) and the lowest number of non-human reads (15,066) of all dataset. The average depth of coverage was 3,713X (standard deviation [SD] = 3,892) for Illumina sequencing and 2,430X (SD = 2,258) for ONT (Additional file 1: Fig. S3), with both technologies showing similar results in amplicon coverage (Additional file 1: Fig. S4, and Additional file 2: Table S3). Consensus sequences covering at least 80% of the genome were obtained for 134 samples sequenced by Illumina (76.6%) and 172 samples sequenced by ONT (98.3%) (Chi-square test, *p* = 2.44 × 10^− 9^). Based on logistic regression models, sequencing success, defined as achieving > 80% of the viral genome, was influenced by sample depth of coverage (ONT, *p* = 6.32 × 10⁻³; Illumina, *p* = 2.6 × 10⁻⁴), Ct (ONT, *p* = 8.91 × 10⁻^6^; Illumina, *p* = 0.019) and patient age (ONT, *p* = 5.17 × 10⁻³; Illumina, *p* = 7.9 × 10⁻³). Notably, in all samples with a Ct ≤ 25, at least 80% of the RSV genome was successfully covered by both sequencing technologies (Additional file 1: Fig. S5 and Fig. S6). Whole-genome lineage classification was concordant in 98.8% of the samples. Only two samples showed misclassification of RSVA/RSVB (Additional file 1: Table S4) and were excluded from further analysis. In these samples, the presence of coinfection was confirmed by RT-qPCR (Fig. 3). Despite the high concordance, there were differences in the observed lineage-defining mutations (Fig. 4). The concordance between the two technologies was 100% for all lineage-defining mutations in lineages A.D.1.8 (*n* = 1), A.D.4 (*n* = 11), A.D.5.1 (*n* = 4), and B.D.4.1.1 (*n* = 5). In the remaining lineages, concordance was also 100% for all mutations except for the following: one mutation for A.D.3 (*n* = 3), two mutations in A.D.1 (*n* = 12), A.D.5.2 (*n* = 20), and A.D.5.3 (*n* = 2), four mutations in B.D.E.1 (*n* = 81), six mutations in A.D.1.5 (*n* = 31), and seven mutations in A.D.3.1 (*n* = 3). Discordance between the two technologies was mainly driven by samples with high Ct, where coverage for those specific positions was not achieved and an undetermined nucleotide was included in the generated consensus sequence. Note that the apparent accumulation of discordance in A.D.3, A.D.3.1, and A.D.5.3 is likely due to the low number of sequences assigned to these lineages (Fig. 4). Notably, a few previously undescribed amino acid substitutions were detected by the two technologies at the same position: the lineage-defining mutations in the B.D.4.1.1 (G:252T, F:103 A and the L:1742 M) and B.D.E.1 (L:715I and L:1742 K) lineages.

For two samples, assigned to A.D.1.5 and A.D.5.2, the discordance in one mutation could not be explained by the low coverage, requiring further investigation. A detailed analysis of the read alignments against the RSV reference genome showed the presence of different nucleotide variants in the same position. To check for an RSVA/RSVB coinfection, an RT-qPCR using the Allplex™ Respiratory Panel 1 kit, which can discriminate between the two subgroups, was performed on the two samples. One of them was positive for both RSVA and RSVB, confirming an RSVA/RSVB coinfection, despite being previously classified exclusively as RSVA by the sequencing technologies. The RT-qPCR result for the other sample showed positivity only for RSVA. To check for the presence of intra-host viral variation, we re-analysed the entire dataset using ViralFlow. ViralFlow provided additional support for a potential coinfection of two RSVA A.D.-like sublineages (A.D.1.5 and A.D.4) in this sample (Fig. 3). Minor variants were not detected in any other sample of the study. Consensus sequences generated in the study are available in GISAID [52].

### Resistance to monoclonal antibodies

To assess the occurrence of amino acid substitutions associated with potential resistance to mAb, we examined the binding sites of palivizumab (F protein, amino acid residues 262–275) and nirsevimab (F protein, amino acid residues 62–69 and 196–212). The mAb target regions were covered in the consensus sequence of 85 out of 87 RSVA samples. Two substitutions were detected in the nirsevimab binding site: F:N63S in 12 of samples (11 in A.D.4 and one in A.D.1.5), and F: I206T in one A.D.3.1. For RSVB, the mAbs target regions were covered in 85 out of 86 RSVB samples, with four substitutions identified in the nirsevimab binding site: 3 sequences carried the F: I206M and F: Q209R substitutions (three B.D.4.1.1 as double mutant F: I206M + F: Q209R) and 79 also carried the F: S211N substitution (78 B.D.E.1 and one B.D.4.1.1 as triple mutant F: I206M + F: Q209R + F: S211N). In addition, one of the B.D.E.1 sequences also carried the F: K68N amino acid change (quadruple mutant F: K68N + F: I206M + F: Q209R + F: S211N) and another the F: K272T substitution in the palivizumab binding site (quadruple mutant F: I206M + F: Q209R + F: S211N + F: K272T).

## Discussion

Genomic surveillance of RSV is of utmost importance for public health, especially for monitoring the potential emergence of nirsevimab-resistant amino acid substitutions of RSV [48]. Here, we successfully adapted and validated a novel RSV genomic surveillance workflow for viral whole-genome sequencing of clinical samples. This workflow consists of a tiling amplicon sequencing protocol for short or long-read sequencing technologies and an in-house bioinformatic pipeline for the analysis of genomic data. Our results demonstrate that the workflow performs equally for both sequencing alternatives, successfully generating consensus sequences and highly concordant lineage classifications in clinical samples from two seasons in the Canary Islands. To date, most of the current RSV genomic surveillance workflows have been developed for a single sequencing technology, either ONT or Illumina [14–19], while only a few have been designed for both sequencing technologies [20–22]. To the best of our knowledge, this is the first bench-to-data analysis workflow developed for RSV whole-genome sequencing that not only provides a direct and comprehensive technological comparison, but also validates the results with clinical samples. Another study employed the ARTIC workflow to compare short- and long-read sequencing technologies [22]; however, their comparative analysis was limited to a small subset of samples (*n* = 35). Our workflow provides optimal sequencing success for samples with Ct < 25. For samples with higher Ct values, it still enables accurate lineage assignment as well as the detection of the amino acid substitutions associated with resistance to mAbs. This high analytical sensitivity is particularly important, as low viral loads are a frequent challenge in clinical practice. It also constitutes the first genomic assessment of the 2022–2023 and 2023–2024 epidemiological seasons of RSV in the Canary Islands and one of the few that have been performed to date in Spain [23, 25].

Two of the 176 analysed clinical samples were classified into different antigenic subgroups by the two technologies, suggesting the presence of coinfections in these samples and prompting further analyses including confirmatory RT-qPCR experiments. Based on the high concordance, the two sequencing technologies were on par to detect the lineage-defining mutations in clinical samples. However, some discordances were observed, especially in lineages A.D.1.5, A.D.5.2, and B.D.E.1. For ONT sequencing, discordance was due to frameshift errors in some of the consensus sequences. For Illumina sequencing, these discrepancies were primarily attributed to a loss of coverage in specific regions, leading to unassigned nucleotides in the consensus sequences. In particular, discordance was accumulated in the L gene, something that can be explained by a drop in coverage in this region in Illumina sequencing. The observed difference between ONT and Illumina in the proportion of consensus sequences covering at least 80% of the genome may also be attributed to this loss of coverage. However, the reason behind this difference in the breadth of coverage between the sequencing solutions is unclear. Despite both technologies employing similar RT-PCR assays, the library preparation protocols differ in crucial reagents such as the reverse transcriptase and the DNA polymerase, which may have affected the overall amplification performance. In addition, the software used in the bioinformatics pipeline differ between the two technologies and may also affect these results. Overall, ONT achieves higher coverage than Illumina sequencing for samples at higher Ct values, suggesting that ONT should be the solution of choice for samples at lower viral load. In two samples, discordance in the lineage assignment could not be fully explained by the loss of coverage, and further analysis revealed the presence of a mixed viral population. Notably, one of the samples with RSVA/RSVB coinfections was obtained from a child with a T-cell lymphoblastic lymphoma who re-tested positive for RSVB only one month after the initial sample was collected. Even though RSVA/RSVB coinfections have been rarely reported so far [53], we have observed them in 3 out of 175 samples with assigned lineage analysed, showing a prevalence of roughly 2% (1.7 every 100 cases) for the studied period. Our findings underscore the importance of continuous monitoring for coinfections in RSV genomic surveillance, particularly in immunocompromised patients, where the RSV burden is high [54, 55]. Additionally, they highlight how having data from both sequencing technologies can enhance the detection and characterization of such cases. Sequencing success of both technologies was influenced by depth of coverage, sample Ct and patient age. While the depth of coverage and Ct are related to the viral load, the effect of patient age on the sequencing success is unclear. Younger children could exhibit weaker immune responses, leading to higher viral loads and lower Ct values, thus increasing the probability of achieving the 80% of the viral genome in our studies. This observation aligns with what has been reported previously [17]. Although the depth of coverage in our analyses reaches several thousand reads per genome, we warn that such a high coverage is not strictly necessary for all analyses. In this particular case, we opted for this high coverage to ensure robust consensus sequence generation and accurate variant calling. However, future studies may prefer a lower sequencing depth to reduce costs and computational requirements achieving similar results.

In terms of computational burden, Kraken2 with the PlusPF database had the highest memory usage requirement, reaching 88.5 GB, whereas all the other tools remained below 5 GB. The complete workflow analysed a batch of 50 Illumina samples in approximately 4 h. By contrast, ONT analysis of the same batch of samples took around 30 min. Although these analyses were conducted on high-end infrastructure, the modest requirements of most tools suggest that the pipeline could be executed on workstations with lower specifications, providing sufficient resources for the taxonomic classification step. Notably, the choice of Kraken2 database strongly influences memory usage, and smaller or custom-built databases may substantially reduce RAM requirements. The modular design enables further scalability to larger datasets when the pipeline is deployed on high-performance computing clusters or cloud infrastructures, supporting its applicability in routine surveillance scenarios.

We had particular interest in identifying the presence of amino acid changes in the antigenic sites of two mAbs, nirsevimab (antigenic site Ø) and palivizumab (antigenic site II), to predict potential resistance to these treatments arising in the community. Within those substitutions found in the nirsevimab binding site, the F: K68N is the only one classified as a nirsevimab neutralisation escape substitution so far [48, 56–60]. In the present study, this substitution was observed in a sample collected during the first epidemiological week of 2023, before the introduction of nirsevimab in the Spanish immunization schedule. Only one sample assigned to the B.D.E.1 lineage was found to carry the F: K272T substitution, which is associated with palivizumab resistance [48, 61, 62].

The specific reference genomes used in this pipeline were chosen based on those employed by Nextclade for clade assignment, ensuring consistency with the widely adopted classification frameworks [50]. We acknowledge that alternative references could lead to small differences in alignment quality, coverage uniformity, or variant calls, particularly for divergent or emerging lineages. However, since the pipeline initially classifies each sample using either a multireference alignment for Illumina or a meta-assembly step for ONT, the subtype assignment remains consistent. Furthermore, the use of only two reference sequences for alignment and consensus generation may also slightly affect the results. Future work could incorporate dynamic reference selection from a broader set of representative genomes, or adopt reference-free or pangenome approaches to reduce potential bias [63].

This study has several limitations. First, the sample collection was limited to patients who required hospital care. Despite this, we showed that the lineages were representative of those transmitted in the community. Second, some mutations may have been missed in some samples due to amplicon dropout, especially in those samples with high Ct values. This issue could be mitigated in the future by adopting a recently published modified version of the tiling amplicon protocol [64].Third, the lack of information on whether patients received mAbs or previous comorbidities limited our ability to analyse RSV sequences in the context of breakthrough infections. Furthermore, we could not distinguish between a reinfection and a prolonged infection when two samples collected from the same patient 30 days apart were assigned to the same lineage. However, our study also has strengths, as each sample was processed using both sequencing technologies, enabling the detection of mixed RSV populations, which was validated through multiple approaches.

## Conclusions

We developed a novel workflow for RSV genomic sequencing and validated it in clinical samples in a real-world setting. Our findings support a high performance with both short- and long-read sequencing technologies. This comprehensive workflow enables robust RSV genomic surveillance and can be readily adapted to either sequencing approaches, accommodating the technical capabilities and requirements of laboratories in routine clinical practice.

## Supplementary Information

Supplementary Material 1.

Supplementary Material 2.

## Data Availability

Consensus sequences in FASTA format have been deposited in GISAID under EPI_SET EPI_SET_251010xw [52]. The code is publicly available and maintained within a GitHub repository (https://github.com/genomicsITER/nf-rsvpipeline).

## Abbreviations

Ct: Cycle threshold
DNA: Deoxyrinonucleic acid
F: Fusion protein gene
G: Attachment glycoprotein gene
IGV: Integrative genome viewer
mAb: Monoclonal antibodies
MSA: Multiple sequence alignment
RNA: Ribonucleic acid
RSVA: Respiratory syncytial virus subgroup A
RSVB: Respiratory syncytial virus subgroup B
RSV: Respiratory syncytial virus
RT-PCR: Reverse transcription – polymerase chain reaction
RT-qPCR: Reverse transcription – quantitative polymerase chain reaction
SD: Standard Deviation
ONT: Oxford Nanopore Technologies
