# Supplementary material for: A bench-to-data analysis workflow for respiratory syncytial virus whole-genome sequencing with short and long-read approaches
Source: Genome Med. 2026 Jan 27;18:9. doi: 10.1186/s13073-025-01597-4 (PMC12837520; doi:10.1186/s13073-025-01597-4)
Supplement: Supplementary file 2 — Supplementary Material 2. [file 13073_2025_1597_MOESM2_ESM.docx]

**A bench-to-data analysis workflow for respiratory syncytial virus whole-genome sequencing with short and long-read approaches**

Adrián Gómez-Del Rosario, Adrián Muñoz-Barrera, Julia Alcoba-Florez, Diego García-Martínez de Artola, Nora Rodríguez-García, Jose Miguel Lorenzo-Salazar, Rafaela González-Montelongo, Carlos Flores, Laura Ciuffreda

**Supplementary Material**

**Table S1.** List of software tools used in the bioinformatic pipeline.

| Step | **Tool** | **Version** | **Illumina** | **Nanopore** |
| --- | --- | --- | --- | --- |
| **Quality control** | FastQC | 2000.11.9 | ✓ |  |
|  | NanoPlot | 1.43.0 |  | ✓ |
| **Taxonomic classification (database)** | Kraken2 (PlusPF) | 2.1.2002 | ✓ | ✓ |
| **Adapter trimming** | fastp | 0.23.2 | ✓ |  |
| **Host reads removal (database)** | Kraken2 (HumanDB) | 2.1.2002 | ✓ | ✓ |
| **Reference selection** | BBMap | 39.08 | ✓ |  |
|  | SPAdes | 3.15.4 | ✓ |  |
|  | BLAST | 2.16.0+ | ✓ |  |
|  | IRMA | 1.2.2000 |  | ✓ |
| **Read alignment** | BWA | 0.7.17-r1188 | ✓ |  |
|  | minimap2 | 2.28-r1209 |  | ✓ |
| **Primer sequence removal** | iVar | 1.3.2001 | ✓ |  |
|  | ARTIC | 1.5.2007 |  | ✓ |
| **Coverage analysis** | MosDepth | 2000.3.3 | ✓ | ✓ |
|  | SAMtools | 1.6 | ✓ | ✓ |
| **Variant-calling and consensus sequence generation** | iVar | 1.3.2001 | ✓ |  |
|  | Medaka | 2.0.0 |  | ✓ |
| **Lineage assignment** | Nextclade | 3.10.2000 | ✓ | ✓ |

**Table S4.** Frequency and percentage of each RSV lineage

| Clade | **Oxford Nanopore Tech. (n=175)** | **Illumina (n=175)** |
| --- | --- | --- |
| A.D.1 | 12 (6.86%) | 12 (6.86%) |
| A.D.1.5 | 31 (17.7%) | 31 (17.7%) |
| A.D.1.8 | 1 (0.57%) | 2 (1.14%) |
| A.D.3 | 3 (1.71%) | 3 (1.71%) |
| A.D.3.1 | 3 (1.71%) | 3 (1.71%) |
| A.D.4 | 11 (6.29%) | 11 (6.29%) |
| A.D.5.1 | 4 (2.29%) | 4 (2.29%) |
| A.D.5.2 | 20 (11.4%) | 21 (12.0%) |
| A.D.5.3 | 2 (1.14%) | 2 (1.14%) |
| B.D.4.1.1 | 5 (2.86%) | 5 (2.86%) |
| B.D.E.1 | 83 (47.4%) | 81 (46.3%) |

**
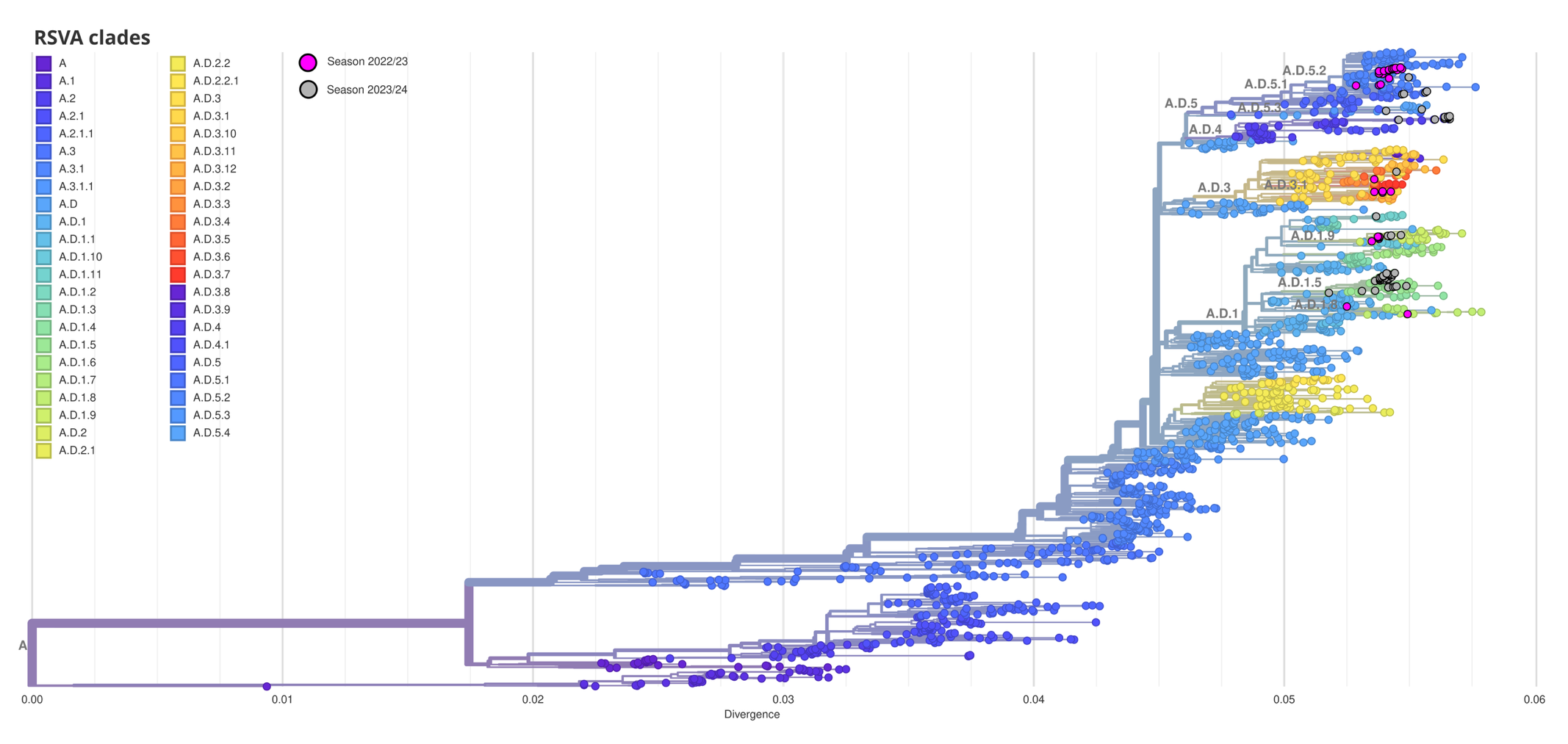
**

**Fig. S1** Phylogenetic tree of the RSVA sequences collected from samples in the Canary Islands from October 2022 to March 2023 (Season 2022/23) and from June 2023 to February 2024 (Season 2023/24). Only sequences obtained from Illumina sequencing are represented.


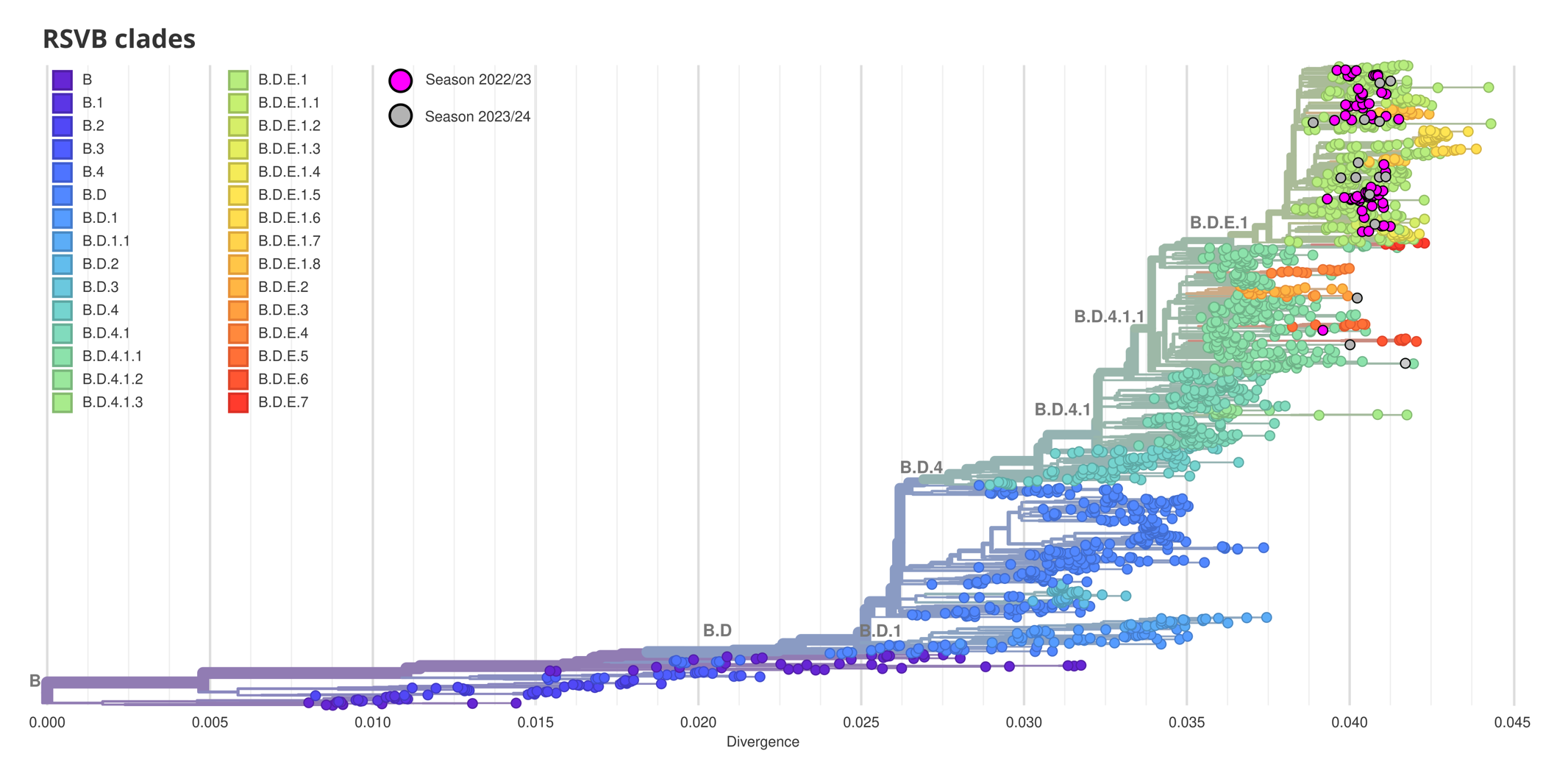


**Fig. S2** Phylogenetic tree of the RSVB sequences collected from samples in the Canary Islands from October 2022 to March 2023 (Season 2022/23) and from June 2023 to February 2024 (Season 2023/24). Only sequences obtained from Illumina sequencing are represented.


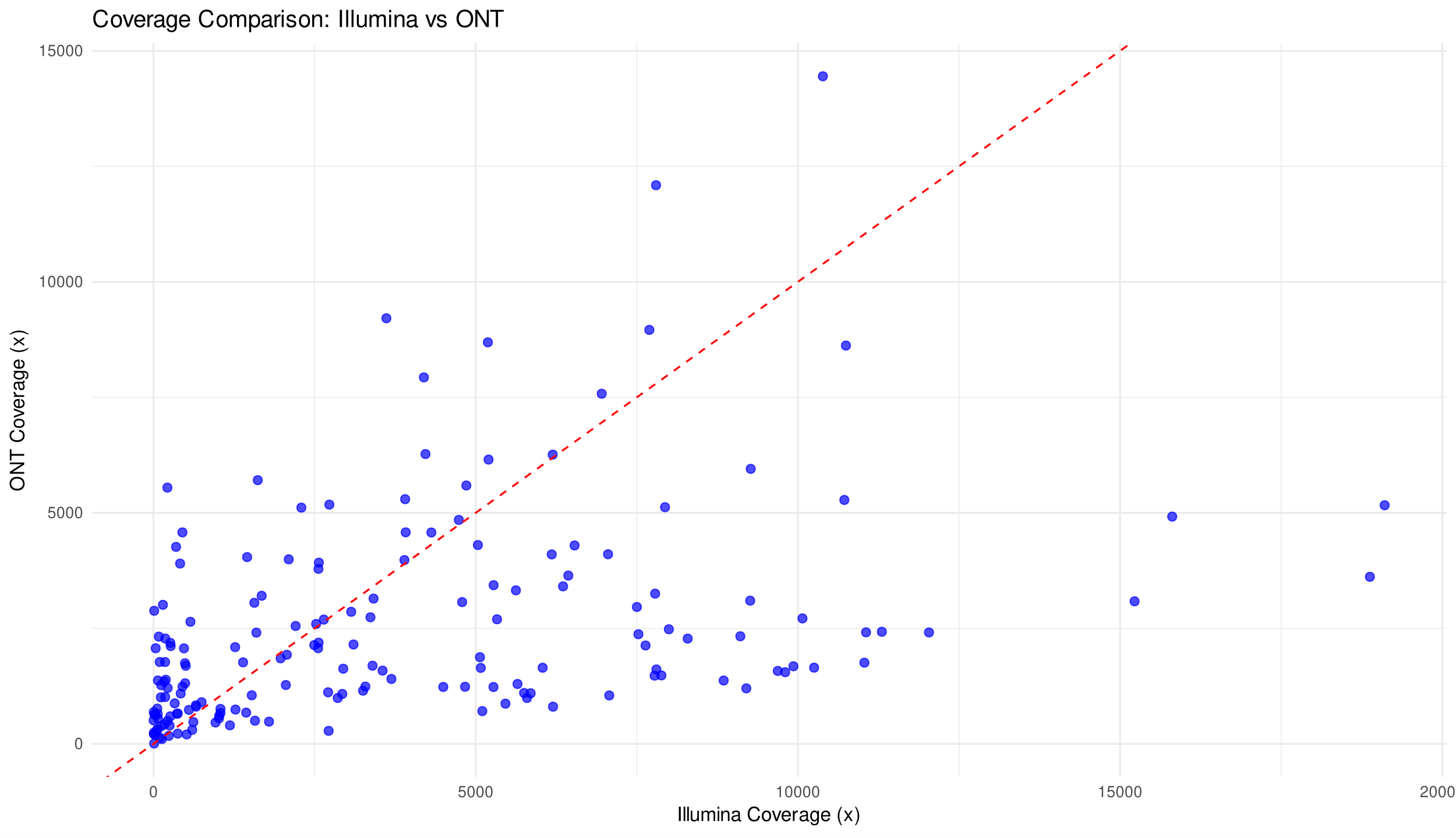


**Fig. S3** Comparison of the coverage obtained using Illumina versus the coverage obtained using Nanopore for all samples.


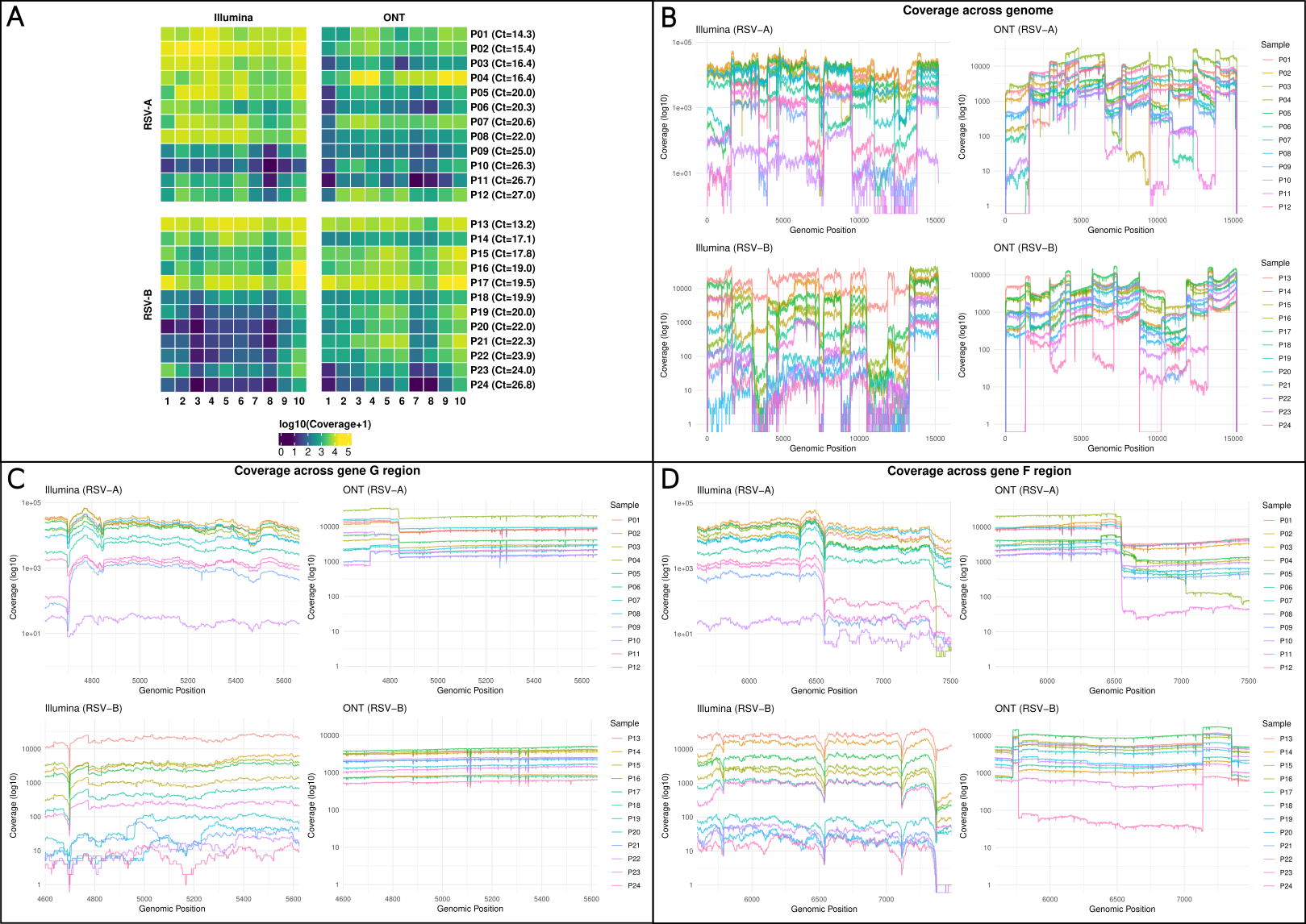


**Fig. S4** **A)** Heatmaps of median amplicon coverage across samples sequenced using Illumina (left) and ONT (right) for samples classified as RSV-A (top) and RSV-B (bottom). Labels in the horizontal axes represent the tiling amplicons used for each antigenic group. **B)** Depth of coverage across genome of RSV-A and RSV-B samples sequenced with Illumina and ONT. **C)** Depth of coverage in gene G region. **D)** Depth of coverage in gene F region. Samples are represented by P1 to P24. These 24 samples were selected based on their Ct values to illustrate coverage across the full range of viral loads (from low to high Ct values). Ct values correspond to those observed qPCR cycle thresholds.


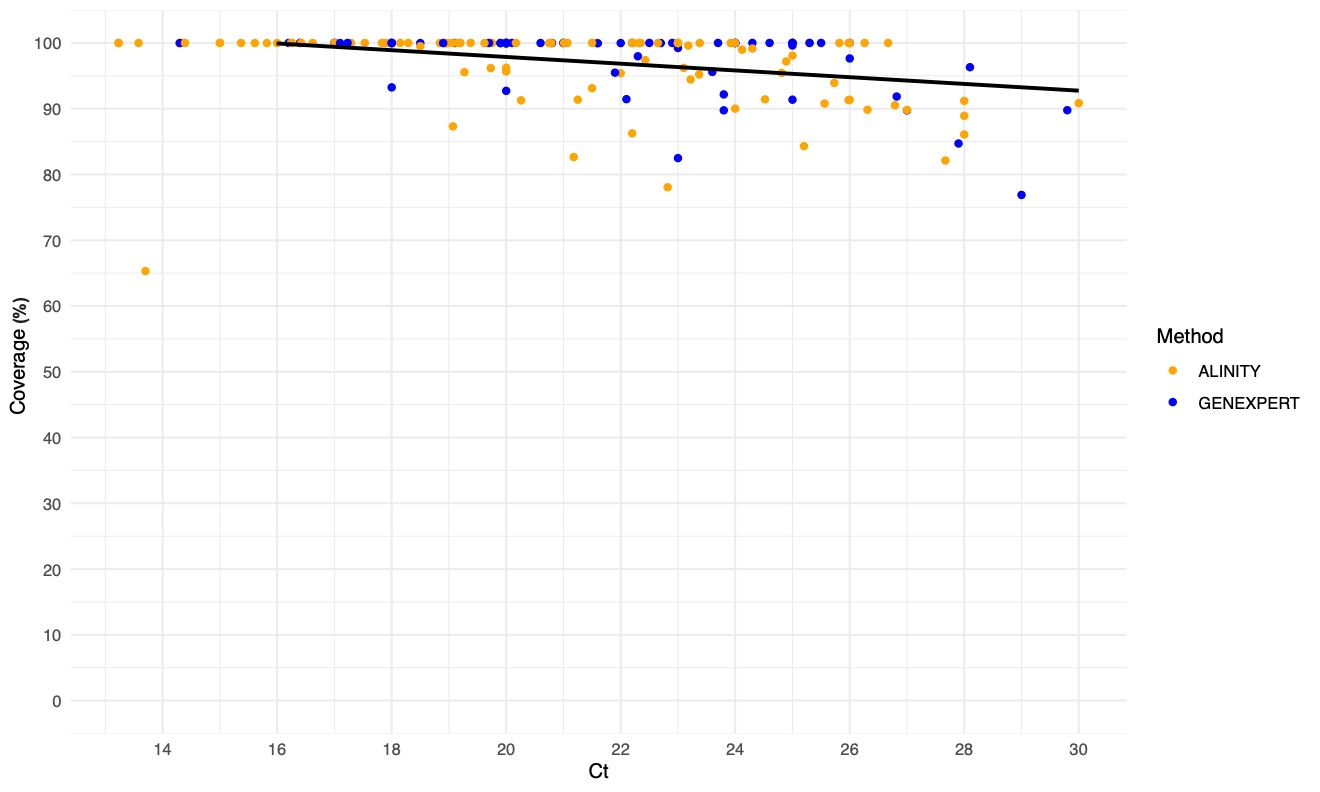


**Fig. S5** Correlation analysis of the breadth of coverage and the cycle threshold (Ct) in RSV samples sequenced with Oxford Nanopore Technologies (*p*=2.81x10^-8^, rho= -0.405).


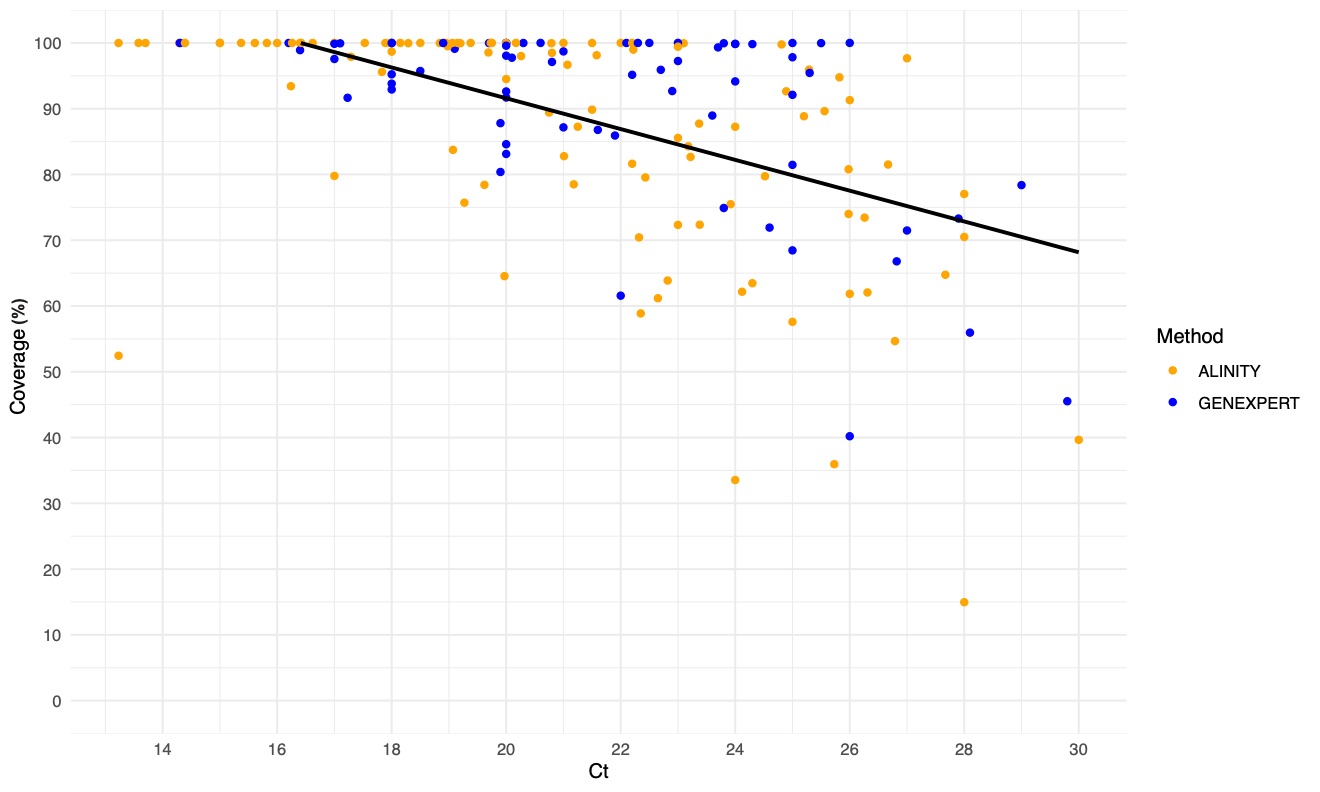


**Fig. S6** Correlation analysis of the breadth of coverage and the cycle threshold (Ct) in RSV samples sequenced with Illumina (*p*=1.56x10^-17^, rho= -0.586).
